# Salt content dependent dielectric properties of pistachios relevant to radio-frequency pasteurization

**DOI:** 10.1038/s41598-019-38987-9

**Published:** 2019-02-20

**Authors:** Seul-Gi Jeong, Sangryeol Ryu, Dong-Hyun Kang

**Affiliations:** 1 Research and Development Division, World Institute of Kimchi, Gwangju, 61755 Republic of Korea; 2 Research Institute for Agricultural and Life Sciences, and Department of Agricultural Biotechnology, Center for Food and Bioconvergence, Seoul National University, Seoul, Republic of Korea; 3 Institutes of Green Bio Science & Technology, Seoul National University, Daehwa-myeon, Pyeongchang-gun, Gangwon-do Republic of Korea

## Abstract

This study was conducted to investigate the effect of salt content during radio-frequency (RF) heating on rate of temperature increase, dielectric properties (DPs), and reduction of pathogens in pistachios. Also, the effect of RF heating on pistachio quality of varying salt content was determined. Pistachios of different salt content (0, 100, and 330 mg sodium/serving) were inoculated with *Salmonella enterica* and treated in a 27.12-MHz RF heater. The RF heating rate increased when salt content was in the range of 0–100 mg sodium/serving, but there were no significant (*P* > 0.05) differences in the rate of temperature rise after salt content reached to 100 mg sodium/serving. Both dielectric constant and dielectric loss factor of pistachios increased with rising salt content. Along with increased salt content, RF treatment time required to reduce this pathogen by 4 log CFU/g decreased first and then remained the same above an upper limit of salt content corresponding to the peak heating rate. RF treatment did not significantly (*P* > 0.05) cause changes in the color and level of lipid oxidation of pistachios. The results of the current study imply that RF heating may be a potential intervention for inactivating foodborne pathogens in pistachios and that the effect of pasteurization is influenced by dielectric loss factor relative to salt content.

## Introduction

Recently, the microbial safety of pistachios has been of great public concern, because salmonellosis has been reported to be associated with pistachios. In 2016, a total of 11 cases of *Salmonella* serovar Montevideo and Senftenberg infections in the United States were traced to contaminated pistachios^1^. Outbreaks of foodborne salmonellosis have caused recalls of pistachio products in 2009 and 2013^2,3^. Strains of *Salmonella* cannot recover on low-moisture foods such as nuts, but may survive for extended periods of time in these environments^4–6^.

In order to prevent microbial growth, decay, and shell staining, pistachios are dried to a moisture level of 5–7% dry basis following harvest and dehulling^7^. Preservation of pistachios through drying based on solar and forced-air drying techniques are commercially available, however these approaches do not assure microbial safety in view of recent outbreaks of salmonellosis from consumption of pistachios^8–10^. Although various decontamination methods, including irradiation, chemical sanitizers, and several thermal processes involving superheated steam and infrared heating, have been proposed to reduce levels of foodborne pathogens in pistachios, these treatments have limitations for commercial application because of poor consumer perception, unhealthful residues, complicated operation, and difficulty of scaling up^11–14^. Therefore, it is necessary to develop new pasteurization processing that can effectively control *Salmonella* in pistachios.

Radio-frequency (RF) heating has emerged as a promising technology due to its faster and more uniform heating than convection and conduction heating^15–19^. RF energy interacts with foods directly and generates heat within the food products because of molecular friction^20^. The effectiveness of RF treatment for inactivating *S*. *enterica* in almonds was compared to convective heating in our previous study^19^. Salazar *et al*.^21^ found the effect of RF treatment on the microbial decontamination of pistachios, and a 3.8-log reduction of *Enterococcus faecium*, namely a surrogate of *Salmonella* spp., was obtained. Dielectric properties (DPs) are essential for preventing undesirable heating leading to product burning or cold spots, because these properties of foods have great effect on their heating rate during RF treatment. The DPs are impacted by sample salt content, moisture content, temperature, density, frequency of the applied alternating electric field, and some other factors^22^. In this study, salt content was chosen as a factor of RF heating for pistachios, since commercially processed pistachios are sold worldwide as light, medium, and highly salted nuts^23^.

In the past 10 years, some studies have been undertaken to verify the impact of salt content on DPs of foods for pasteurization, drying, thawing, and disinfestation^24–28^. Ling *et al*.^25^ reported that the DPs of pistachio kernel samples increased with increasing salt content. The similar trends in salmon and sturgeon caviar were also found by Al-Holy *et al*.^29^. However, no comprehensive report has elucidated the effect of salt content on the rate of temperature rise, DPs, and microbial inactivation in foods during RF treatment. Also, the quality changes occurring during RF heating at different salt contents have not been reported. In order to optimize treatment conditions for RF heating without affecting product quality, an investigation of heating rates of food products which depend on salt content is needed.

The overall objective of current study was to examine the influence of salt content on rate of temperature increase and DPs of pistachios. The effects of RF heating on inactivation of *Salmonella enterica* and quality factors of pistachios with different salt levels, including color values, acid values, and peroxide value, were investigated.

## Results

### Average temperature-time histories of pistachios with varying salt contents

**Figure 1:**
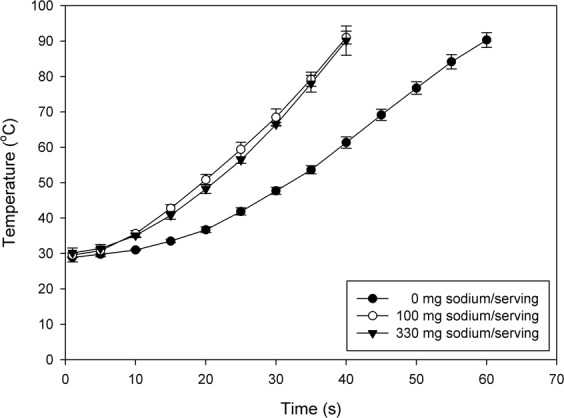
Temperature curves of pistachios with salt contents of 0 mg (●), 100 mg (◯), and 330 mg sodium/serving (▼) during RF heating.

Temperature histories of pistachios with salt content range of 0–330 mg sodium/serving during RF treatment are depicted in Fig. 1. Temperature rapidly rose as treatment time increased at the same salt content. The heating rate of salted pistachios was much higher than that of non-salted pistachios, but significant differences (*P* < 0.05) above a salt content of 100 mg sodium/serving were not observed. After 40 s of RF heating, the temperature of salted pistachios in the range of 100–330 mg sodium/serving reached ca. 90 °C. To raise the temperature of non-salted pistachios to 90 °C, a maximum of ca. 60 s was required.

### Influence of salt content on DPs and penetration depth of pistachios

Table 1 shows the DPs and penetration depth of pistachio nuts of varying salt contents at 27.12 MHz. Salt content had a significant (*P* < 0.05) positive effect on both the dielectric constant and dielectric loss factor of pistachio kernels. Greater dependence on salt content was observed in dielectric loss factors compared to dielectric constants of the samples. The salt content of pistachios were negatively correlated to the penetration depth of RF energy. Increasing salt content from 0 to 330 mg sodium/serving reduced penetration depth by about 35 cm at 27.12 MHz.

**Table 1:** DPs and power penetration depths of pistachios of different salt content at 27.12 MHz^a^.

Salt content (mg sodium/serving)	DPs		Penetration depth (cm)
	Dielectric constant (ε′)	Dielectric loss factor (ε″)	
0	10.37 ± 0.64 c	5.33 ± 0.22 c	53.92 ± 3.90 a
100	15.34 ± 0.20 b	15.83 ± 0.29 b	31.29 ± 2.04 b
330	23.78 ± 0.38 a	42.83 ± 2.20 a	19.02 ± 2.23 c

^a^Data are means ± standard deviations from three replications. Values in the same column followed by different letters are significantly different (*P* < 0.05).

### Relationships between salt content, heating rate, and dielectric loss factor of pistachios

**Figure 2:**
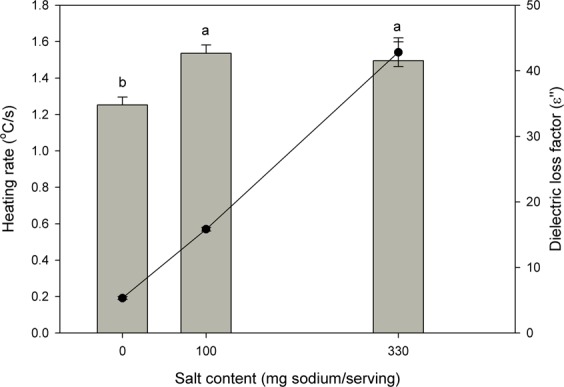
Tripartite relationships between the salt content, heating rate, and dielectric loss factor of pistachios during RF heating.

The results of salt contents, the rate of temperature rise, and dielectric loss factor of pistachios were depicted in Fig. 2 for interrelationship analysis. As presented in Table 1, the dielectric loss factor was proportional to salt content. The rate of temperature increase was dependent on salt content within the range of 0–100 mg sodium/serving as presented in Fig. 1. The overall trends between the rate of temperature rise and the dielectric loss factor were comparable to those between the rate of temperature rise and salt content. The heating rate remained stable when the dielectric loss factor further increased (above 15.83) in pistachios.

### Bactericidal effects by salt content in pistachios

**Figure 3:**
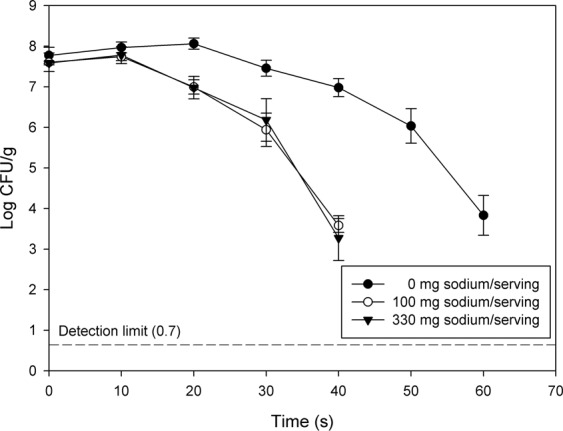
Survival curves for *Salmonella enterica* in pistachios during RF heating as affected by salt level.

Reduction of *S*. *enterica* in pistachios with different salt content after RF heating is illustrated in Fig. 3. Populations of this pathogenic bacterium reduced as treatment time increased. Although no significant (*P* > 0.05) differences in cell numbers after 10 s treatment within all tested levels of salt content were revealed, *S*. *enterica* levels were reduced by about 0.61 log after 20 s of RF heating in salted pistachios. Log reductions of 1.66 and 1.41, respectively, were observed in pistachios with salt contents of 100 and 330 mg sodium/serving after RF heating for 30 s. The reductions of *S*. *enterica* in non-salted pistachios were much lower than those achieved in salted pistachios. However, after the salt content reached to 100 mg sodium/serving, the bactericidal effects of RF heating remained the same. At 100 mg and 330 mg sodium/serving, 4-log reductions were achieved for *S*. *enterica* after 40 s of treatment. Populations of this pathogen in pistachios decreased by same levels when treated for 60 s at 0 mg sodium/serving.

### Resuscitation of heat-injured cells

Levels of heat-injured *S*. *enterica* cells in pistachios following RF heating are presented in Table 2. Slightly lower reductions of this pathogen were induced by the procedure involving the recovery step than by direct plating on selective medium. Especially, after the maximum RF treatment, there were differences between levels of cells counted on the selective medium (XLD) and the recovery medium (OV-XLD), indicating sublethally injured *S*. *enterica* cells in pistachios. However, over the entire span of treatment times, statistically significant differences (*P* < 0.05) were not observed between surviving populations a result of heat-injured cell resuscitation in pistachios of varying salt content.

**Table 2:** Levels of surviving populations and populations including sublethally injured *Salmonella enterica* in pistachios on culture media after RF treatment^a^.

Salt content (mg sodium/serving)	Treatment time (s)	Population (log CFU/g)	
		XLD^b^	OV-XLD
0	0	7.77 ± 0.20 ABa	7.82 ± 0.15 Aa
	10	7.97 ± 0.13 ABa	8.02 ± 0.08 Aa
	20	8.06 ± 0.14 Aa	8.07 ± 0.18 Aa
	30	7.46 ± 0.20 BCa	7.53 ± 0.18 ABa
	40	6.98 ± 0.22 Ca	7.06 ± 0.17 Ba
	50	6.03 ± 0.42 Da	6.23 ± 0.31 Ca
	60	3.38 ± 0.49 Ea	3.97 ± 0.71 Da
100	0	7.60 ± 0.23 ABa	7.74 ± 0.20 Aa
	10	7.74 ± 0.10 Aa	7.83 ± 0.09 Aa
	20	6.99 ± 0.18 Ba	7.21 ± 0.35 Aa
	30	5.94 ± 0.41 Ca	6.01 ± 0.34 Ba
	40	3.58 ± 0.17 Da	3.74 ± 0.48 Ca
330	0	7.59 ± 0.05 ABa	7.58 ± 0.06 ABa
	10	7.78 ± 0.21 Aa	7.79 ± 0.24 Aa
	20	6.98 ± 0.28 Ba	7.04 ± 0.22 Ba
	30	6.18 ± 0.52 Ca	6.58 ± 0.52 Ca
	40	3.27 ± 0.55 Da	3.56 ± 0.45 Da

^a^Data are means ± standard deviations from three replications. Means in the same column followed by the different capital letter are significantly different (*P* < 0.05). Values with the same small letter in the same row are not significantly different (*P* > 0.05).

^b^XLD, xylose lysine desoxycholate; OV-XLD, overlay XLD agar on TSA.

### Effect of RF heating on product quality within different salt range

The color values and oxidative rancidity of pistachios of various salt content after RF treatment for time intervals required to decrease *S*. *enterica* by 4 log CFU/g are shown in Tables 3 and 4. There were no significant (*P* > 0.05) differences in L^*^, a^*^, and b^*^ values between treated and untreated pistachios regardless of salt content. The acid value and peroxide value of RF-treated samples were also not significantly (*P* > 0.05) different from values of the untreated control. Even though these quality parameters changed slightly depending on RF treatment at varying salt content, statistically significant differences were not observed among all tested samples (*P* > 0.05).

**Table 3:** Color values of RF-treated pistachios of different salt content^a^.

Parameter and treatment type	Salt content (mg sodium/serving)		
	0	100	330
L* (lightness)			
None	53.24 ± 1.85	50.29 ± 1.64	48.43 ± 0.46
RF treated	53.42 ± 0.77	49.32 ± 0.87	48.73 ± 0.43
a* (redness)			
None	2.79 ± 0.48	5.47 ± 0.51	8.51 ± 0.38
RF treated	3.47 ± 0.47	5.72 ± 0.23	8.13 ± 0.21
b* (yellowness)			
None	13.69 ± 1.13	6.01 ± 1.52	0.40 ± 0.02
RF treated	13.60 ± 2.63	6.40 ± 0.42	0.43 ± 0.04

^a^Data are means ± standard deviations from three replications. No values are not significantly different (*P* > 0.05) within a column per parameter.

**Table 4:** Acid values and peroxide values of RF-treated pistachios of different salt content^a^.

Parameter and treatment type	Salt content (mg sodium/serving)		
	0	100	330
**Acid value (%)**			
None	0.69 ± 0.10	0.73 ± 0.09	0.86 ± 0.12
RF treated	0.72 ± 0.05	0.70 ± 0.06	0.83 ± 0.40
**Peroxide value (meq/kg)**			
None	0.71 ± 0.13	0.90 ± 0.20	0.93 ± 0.02
RF treated	0.67 ± 0.07	1.01 ± 0.08	0.93 ± 0.55

^a^Data are means ± standard deviations from three replications. No values are not significantly different (*P* > 0.05) within a column per parameter.

## Discussion

In recent years, there has been increasing acceptance of RF heating for foods with low thermal conductivity. Although RF heating has been reported as an alternative food processing technology because of internal heating as a result of the direct interaction between the food material and electromagnetic waves^17,30–32^, to date, no research has been conducted to inactivate pathogenic bacteria on pistachios of different salt content using RF treatment. In this study, salt content of pistachios affected pathogen reductions. In non-salted pistachios, 60 s was required to decrease *S*. *enterica* by 4 log CFU/g, but only 40 s was required in salted pistachios. There was an upper limit at which rising salt content resulted in a determination of RF heating time required to achieve 4-log reduction of *S*. *enterica*. Since non-thermal effects of RF energy on foodborne pathogens are not detected^33,34^, the upper limit in antimicrobial effect was caused by a salt-dependent rate of temperature increase.

RF heating is influenced by DPs which consist of the dielectric constant and dielectric loss factor. The dielectric constant reflects the ability of a substance to conserve electric energy and the polarizing effect from an alternating electrical field, which is related to how easily the dielectric material is polarized. The dielectric loss factor is a measure of the dissipation of electric energy in the form of heat; namely, how the energy from an applied electrical field is converted to heat^22,35^. The influence of salt content on DPs of processed pistachios was verified by our data which suggested small increase in the dielectric constant and drastic increase in the dielectric loss factor. The dissolved ions coming from salt could enhance electrophoretic migration in the food material and the overall DPs, especially dielectric loss factor^36^. Ling *et al*.^25^ also found similar patters at various salt levels. Therefore, it is extremely necessary to take into account the DPs of foods which depend on their salt content in order to design successful RF heating processes.

In general, an inverse relationship between salt content and penetration depth has been reported^26,29^. This result is in good agreement with our data which indicated that penetration depth decreased significantly (*P* < 0.05) as salt content of pistachios increased. Since the smaller penetration depth values of salted pistachios could result in more surface heating and a lower heating rate, the thickness of pistachios should be smaller than penetration depth of the RF heating system. In this study, RF energy had a penetration depth of more than 19 cm, which was enough to penetrate pistachios contained in the polypropylene jar which has a thickness of 4 cm. The penetration depth values shown in Table 1 can be utilized as a practicable database for determining the amount of pistachios in sample containers during RF treatment.

The influence of DPs on heating rate during RF treatment corresponded to the equation $P=2\pi f\,{V}^{2}{\varepsilon }_{0}\varepsilon ^{\prime\prime}$, where *P* is the electrical power transferred to the dielectric material (e.g. food) as heat, *f* is the frequency, *V* is the electric field strength, *ε*_0_ is the dielectric constant of a vacuum (8.85 × 10^−12^ F/m), and *ε*″ is the dielectric loss factor of the food sample^20^. According to the equation, heat generation is in proportion to the frequency, the electric field strength, and the dielectric loss factor. In the present research, we investigated only the impact of the dielectric loss factor of pistachios relative to salt content during RF treatment, due to fixed constant values of frequency and electric field strength at 27.12 MHz and 0.3 kV/cm, respectively. Our results were consistent with the equation; higher dielectric loss factors caused by higher salt content produced greater heat generation. However, further increasing dielectric loss factor over 15.83 maintained the heating rate, upper limit of salt content was 100 mg sodium/serving for RF treatment in pistachios. This result is consistent with a previous study which proposed that when the dielectric loss factor exceeded an optimum level, current leakage took place through the sample led to preventing continuous increase of temperature^37,38^. Our previous research also found that there is a threshold level of dielectric loss factor for achieving maximum effectiveness of RF heating^32^.

It is essential to consider the effect of sublethally injured cells which are as critical as their normal counterparts because of their ability of recovering pathogenicity under suitable environmental conditions^39,40^. Therefore, in this study, a resuscitation step for heat-injured pathogens on nonselective medium was conducted according to overlay method, since injured cells cannot grow on selective medium. After RF heating at various salt contents, statistically significant differences between populations of uninjured and injured cells on pistachios were not observed (*P* > 0.05). These results imply that RF heating effectively reduced *S*. *enterica* on pistachios without producing appreciable numbers of heat-injured cells.

In addition, there is a need to examine quality changes induced by RF heating for validation of feasible application of this pasteurization processing. Due to the potential for quality indicators to be influenced by high temperature during RF treatment, we focused our attention on measurement of color values, acid values, and peroxide values of pistachios. After the maximum treatment for pistachios of varying salt content, all tested quality parameters did not show statistically significant differences from those of the control (*P* > 0.05). Other research investigations also found that the quality of agricultural commodities was not affected by RF heating. In a study performed by Gao *et al*.^41^, RF heated almonds maintained levels of color and lipid oxidation. Zheng *et al*.^42^ reported that RF treatment generated no loss in color, water activity, moisture content, ash, protein, starch, fat, or fatty acids.

Although RF heating offers more uniform heating due to both longer wavelength and deeper penetration depth compared to microwave heating, non-uniform temperature distribution can be a problem for industrial-scale RF heating. RF heating uniformity could be enhanced by utilizing a form of the continuous mixing process^43,44^. A screw conveyor combined with an industrial RF heating system could generate a uniform electric field resulting from changes in the positions of the food sample, especially in cold spots and hot spots and the bottom and top layers^45,46^. RF heating on an industrial scale for inactivating pathogenic bacteria in pistachios should be founded on proper treatment times which depend on salt content, since overheating or insufficient heating during RF treatment can cause quality loss of food products or enable survival of microorganisms. Therefore, in order to assure uniform commercial sterilization and minimize quality changes, further studies relevant to inactivation modeling of foodborne pathogens are required.

Given our results, RF heating can be applied to inactivate *S*. *enterica* in pistachios of varying salt content without affecting quality deterioration. The effect of pathogen reduction is affected by DPs of food products which depend on its salt content. The results obtained from the present study will be useful in modeling the reaction of pistachios to RF energy at certain salt contents, and moreover, in applying commercial-scale RF pasteurization. With a thorough understanding of the effect of other factors as well as salt content on the rate of temperature increase in pistachios, RF heating could be expanded to practical industrial scale.

## Methods

### Preparation of inoculum

Three serovars of *S*. *enterica*, namely, Enteritidis PT 30 (ATCC BAA-1045), Senftenberg (KVCC 0590), and Typhimurium (ATCC 19585) were obtained from our personal culture collection. Inoculum was prepared using the procedure described previously^19,47^, with some modifications. Cultures were grown individually in 30 ml of tryptic soy broth (TSB; BD Difco, New Jersey, United States) at 37 °C for 24 h, which was followed by a subsequent loopful (ca. 10 μl) transfer into 30 ml of TSB and overnight incubation at 37 °C. One-milliliter quantities of each strain was equally distributed between three tryptic soy agar (TSA; BD Difco) plates, spread plated, and incubated at 37 °C for 24 h to produce a bacterial lawn. For cell harvesting, approximately 9 ml of sterile 0.2% peptone water (PW; BD Difco) was added to each plate, then a sterile cotton swab was used to loosen the bacterial lawn. Following collection into a sterile container, cell suspensions of all strains of *S*. *enterica* were combined at an equal volume (9 ml each) for a final concentration of approximately 10^9^ CFU/ml.

### Preparation of sample with different salt contents

Three types of commercial pistachios (The Wonderful Co., California, United States) were purchased at a local market (Seoul, Republic of Korea). The moisture, mineral, and total lipid content were determined using a halogen moisture analyzer (MB45; Ohaus, New Jersey, United States), a muffle furnace (WiseTherm F-27; Daihan Scientific, Wonju, Republic of Korea), and a Soxhlet extractor (Det-gras N 4002842; J.P. Selecta, Barcelona, Spain), respectively, since the DPs are dependent on these ingredients. According to the nutrition facts label and the actual measured values (Table 5), these processed pistachios contained the same ingredients apart from three levels of salt: 0, 100, and 330 mg sodium/serving.

**Table 5:** Composition of commercial pistachios with three different salt levels^a^.

Ingredient (%)	Salt content (mg sodium/serving)		
	0	100	330
Moisture	3.49 ± 0.35	4.08 ± 0.38	3.75 ± 0.03
Mineral	3.85 ± 0.07	3.55 ± 0.34	3.89 ± 0.26
Total lipid	45.15 ± 1.51	45.49 ± 1.09	44.93 ± 2.05

^a^Data are means ± standard deviations from three replications. No values are not significantly different (*P* > 0.05) within a row.

### Sample inoculation

Pistachio samples (400 ± 1 g) were weighed into blender bags (SCT-6090A; Labphas, Inc., Quebec, Canada), and 25 ml of culture cocktail was added. The inoculated samples were gently mixed to guarantee even distribution of the culture and dried for 24 h inside a biosafety hood (24 ± 2 °C) with the fan running until the moisture content of pistachios reached that of uninoculated control (ca. 4.0% d.b.). The moisture contents of inoculated and uninoculated samples were measured using the halogen moisture analyzer. The inoculated pistachio samples with a final cell concentration ranged approximately from 10^7^ to 10^8^ CFU/g were used in pasteurization experiment.

### Experimental equipment

The RF heating and DPs measurements were carried out in an apparatus described previously^32,48^. The RF heating system was constructed by Dong Young Engineering Co. Ltd. (Gimhae, Republic of Korea). The lab-scale 9-kW, 27.12-MHz RF heater generated a RF electric field, and its cavity consisted of two parallel rectangular electrodes (30.0 × 35.0 cm), which were 7.0 cm apart. The DPs measurement system was composed of a precision impedance analyzer (4294 A; Keysight Technologies, California, United States), a liquid test fixture (16452 A; Keysight Technologies), and a port extension cable (16048 G; Keysight Technologies). For calculation of DPs of the samples, the precision impedance analyzer measured electrical parameters of the sample.

### RF heating treatment

A 25 g sample of inoculated pistachios was transferred into a polypropylene jar (NALGENE 2118-0002; diameter, 4.5 cm; height, 4.0 cm; Thermo Fisher Scientific, Massachusetts, United States) and placed on the center of the bottom electrode. RF energy was applied to pistachios and heated to 90 °C in order to prevent further product damage while maximizing the efficacy of pasteurization. During RF treatment, a fiber optic temperature sensor with an accuracy of ±1 °C (FOT-L; FISO Technologies Inc., Quebec, Canada) coupled with a temperature signal conditioner (TMI-4; FISO Technologies Inc.) was used to measure real-time temperature of samples. The sensor was inserted into the center of a pistachio kernel located in the middle through a perforated hole, and the temperature was manually measured at 5 s intervals. The fiber optic probe was not electronically active and did not affect the temperature profile of the treated pistachios. The heating rate was calculated by dividing the changes in temperature by treatment time.

### DPs measurement

**Figure 4:**
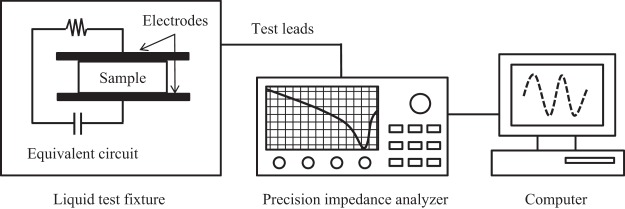
Schematic diagram of DPs measurement system at Seoul National University (Seoul, Republic of Korea).

The DPs of samples were tested according to the procedure described by Jeong and Kang^32^ (Fig. 4). Since pistachio kernels have an uneven shape and cannot make complete contact with the flat electrode, the samples were ground for using this parallel plate method^25,49^. Briefly, the precision impedance analyzer was manually calibrated with a 100 Ohm resistor (04294-61001; Keysight Technologies), and then a predetermined maximum amount of ground sample corresponding to the sample capacity was placed into the test fixture in order to investigate only the effect of salt content on DPs due to a fixed density value of 0.42 g/ml. The sample capacitance, resistance, and air capacitance were measured automatically at 27.12 MHz and ambient temperature (24 ± 2 °C). The DPs of pistachios, especially dielectric constant and dielectric loss factor, were calculated in accordance with ASTM D150. The penetration depth was calculated from the dielectric constant and dielectric loss factor using the following equation:

$${d}_{p}=\frac{c}{\omega \sqrt{2\varepsilon ^{\prime} [\sqrt{1+{(\frac{\varepsilon ^{\prime\prime} }{\varepsilon ^{\prime} })}^{2}-1}]}}$$

where *d*_*p*_ is the penetration depth, *c* is the velocity of light in a vacuum (3 × 10^8^ m/s), *ω* is the angular frequency (2πf), ε′ is the dielectric constant, and ε″ is the dielectric loss factor.

### Bacterial enumeration

At selected time intervals, treated samples (25 g) were instantly placed in sterile blender bags and diluted with 100 ml of 0.2% sterile PW, and then homogenized for 2 min with a stomacher (easyMIX; AES Chemunex, Bruz, France). One-milliliter stomached sample aliquots were 10-fold serially diluted in 9 ml of sterile 0.2% PW, and 100 μl of selected diluents were plated onto xylose lysine desoxycholate (XLD; Oxoid, Hampshire, United Kingdom) agar. Colonies were enumerated on XLD plates after incubation at 37 °C for 24 h. Random black colonies were selected from the plates and confirmed by serological agglutination test using the *Salmonella* latex test kit (FT0203; Oxoid).

### Enumeration of injured cells

For enumeration of injured *S*. *enterica* cells, TSA was used as a nonselective agar to repair sublethally heat-injured pathogens using the overlay method^50^. Appropriate diluents (100 μl) were plated on TSA and incubated for 2 h at 37 °C to enable damaged bacteria to resuscitate, and then about 8 to 10 ml of XLD tempered to 45 °C was poured onto the plates. All agar plates were solidified and incubated at 37 °C for an extra 22 h, and the characteristic *Salmonella* colonies were counted.

### Color, acid value, and peroxide value measurement

Quality measurement was performed to verify the effect of RF treatment on the quality changes. Kernel skin color of treated and untreated pistachios were assessed by measuring L* (lightness), a* (redness), and b* (yellowness) values using a chromameter (CR-400; Konica Minolta, Tokyo, Japan) at identical locations. Color values were quantified by CIELAB space with illuminant C and 2° standard observer. In order to evaluate how specific indicators of lipid oxidation varied over RF treated pistachios compared to those of untreated samples, acid value and peroxide value were measured by extracting and titrating the oil from the samples, as described in the standard methods Cd 8b-90 and Cd 3d-63 of the American Oil Chemists’ Society.

### Statistical analysis

All experiments were conducted in triplicate. All data were analyzed using the Statistical Analysis System software (SAS Institute, North Carolina, United States). Analysis of variance procedure was used to determine the significant differences in the treatments at *P* < 0.05 using Duncan’s multiple-range test.
